# Antibacterial and Antibiofilm Activities of Psychorubrin, a Pyranonaphthoquinone Isolated From *Mitracarpus frigidus* (Rubiaceae)

**DOI:** 10.3389/fmicb.2018.00724

**Published:** 2018-04-13

**Authors:** Ari S. O. Lemos, Lara M. Campos, Lívia Melo, Maria C. M. R. Guedes, Luiz G. Oliveira, Thiago P. Silva, Rossana C. N. Melo, Vinícius N. Rocha, Jair A. K. Aguiar, Ana C. M. Apolônio, Elita Scio, Rodrigo L. Fabri

**Affiliations:** ^1^Bioactive Natural Products Laboratory, Department of Biochemistry, Institute of Biological Sciences, Federal University of Juiz de Fora, Juiz de Fora, Brazil; ^2^Laboratory of Glycoconjugate Analysis, Department of Biochemistry, Institute of Biological Sciences, Federal University of Juiz de Fora, Juiz de Fora, Brazil; ^3^Laboratory of Cellular Biology, Department of Biology, Institute of Biological Sciences, Federal University of Juiz de Fora, Juiz de Fora, Brazil; ^4^Department of Veterinary Medicine, Faculty of Medicine, Federal University of Juiz de Fora, Juiz de Fora, Brazil; ^5^Laboratory of Bacterial Physiology and Molecular Genetics, Department of Parasitology, Microbiology and Immunology, Institute of Biological Sciences, Federal University of Juiz de Fora, Juiz de Fora, Brazil

**Keywords:** psychorubrin, antibacterial activity, MRSA, *Mitracarpus frigidus*, biofilm control

## Abstract

Psychorubrin, a natural pyranonaphthoquinone found in different plants, has become an interesting compound in the search for new antimicrobial therapeutic agents. Here, we investigated the potential antagonistic activity of psychorubrin against planktonic and biofilm bacteria. First, psychorubrin was tested against several Gram-positive and Gram-negative bacteria strains by a broth microdilution susceptibility method. Second, bacterial killing assay, bacterial abundance, and membrane viability were evaluated. The nucleotide leakage assay was used to verify membrane destabilization while antibiofilm activities were analyzed by the effect on established biofilm, static biofilm formation, isolation of biofilm matrix assay and scanning electron microscopy. In parallel, the combinatorial effect of psychorubrin and chloramphenicol was evaluated by the checkerboard method. Psychorubrin was active against Gram-positive bacteria, showing rapid time-dependent kinetics of bacterial killing, amplified nucleotide leakage, and greater activity against the methicillin-resistant species (MRSA) *Staphylococcus aureus* 33591 and 33592 and *Staphylococcus pyogenes* 10096. Psychorubrin also interfered with the composition of the biofilm matrix by reducing the total content of carbohydrates and proteins. A synergic effect between psychorubrin and chloramphenicol was observed for *S. aureus* 33592 and *S. pyogenes* 10096 while an additive effect was detected for *S. aureus* 33591. Our findings demonstrate, for the first time, an antagonistic activity of psychorubrin against bacteria not only in their planktonic forms but also in biofilms, and identify bacterial membranes as primary targets for this compound. Based on these observations, psychorubrin has a good potential for the design of novel antimicrobial agents.

## Introduction

*Staphylococcus aureus* is a major human pathogen that can cause varied diseases, ranging from minor skin infections to severe systemic diseases such as septicemia and pneumonia (reviewed in Kobayashi et al., 2015). Moreover, new resistant strains of *S. aureus* have arisen with tentative treatments of the pathologies caused by this bacterium (Choo, 2017; Oestergaard et al., 2017). The bacteremia caused by methicillin-resistant *S. aureus* (MRSA), for example, is associated with increased morbidity and mortality in adults and its frequency has become greater in hospital institutions (Wilson et al., 2017). In 2004, the Center for Disease Control showed that MRSA proportion was higher than 50% in intensive care unit patients, considering the hospitals that integrated the National Nosocomial Infections Surveillance System [NNIS] (2004). However, multidrug-resistant organisms have not emerged only in the hospital environment. In recent years, due to the exacerbated use of antibiotics, MRSA has also been increasingly found in community-onset infections (Choo, 2017). MRSA is widely prevalent worldwide, with rates highest than 50% reported in North and South America, Asia, and Malta (Stefani et al., 2012). Diverse antimicrobial classes including the *β*-lactams, the glycopeptides, and the fluoroquinolones have been recognized as a major problem in public health because of their resistance (Mai et al., 2017).

*Streptococcus pyogenes* (group A streptococcus) is another common human pathogen. Although most infections caused by *S. pyogenes* are benign and have short duration, this agent may cause late sequelae such as post-streptococcal acute glomerulonephritis, rheumatic fever, streptococcal toxic shock syndrome, necrotizing fasciitis, and other localized or systemic infections, which may present a fulminate evolution (reviewed in Carapetis et al., 2005; Imohl et al., 2017). The global impact of invasive *S. pyogenes* disease is high with, at least, 663,000 new cases and 163,000 deaths worldwide each year (Carapetis et al., 2005).

Although resistant bacteria in their free-living forms have been a major concern for the health system, when they congregate in large numbers to form a biofilm, the problem is even bigger (Ribeiro et al., 2016). In fact, biofilms, densely packed communities of microbial cells growing in a living or inert surface and surrounded by a self-produced polymeric matrix, requires much higher doses of antibiotics (10–1000 times) for bacterial killing and can lead to chronic and persistent infections (Mishra and Wang, 2017). This occurs because biofilm protects bacteria against several physicochemical aggressions, including ultraviolet light, heavy metals, acidity, modulation in hydration or salinity, and phagocytosis (Lebeaux et al., 2014). Thus, bacteria growing in a biofilm are highly resistant to antibiotic treatment and host immune defense and, once established, a biofilm becomes difficult to eradicate (Wu et al., 2015).

Because antimicrobial resistance and biofilm formation are a global public health challenge, there is a striking need of development of new biologically active molecules against multidrug-resistant bacteria and the adoption of medicinal crude extracts of plants to treat infectious diseases (Subramani et al., 2017). In this context, our group has been studying different potential bioactive antibacterial compounds (Pinto et al., 2017a,b), including a natural product termed psychorubrin, a napthoquinone commonly found in a variety of plants (Fabri et al., 2009, 2012).

**FIGURE 1:**
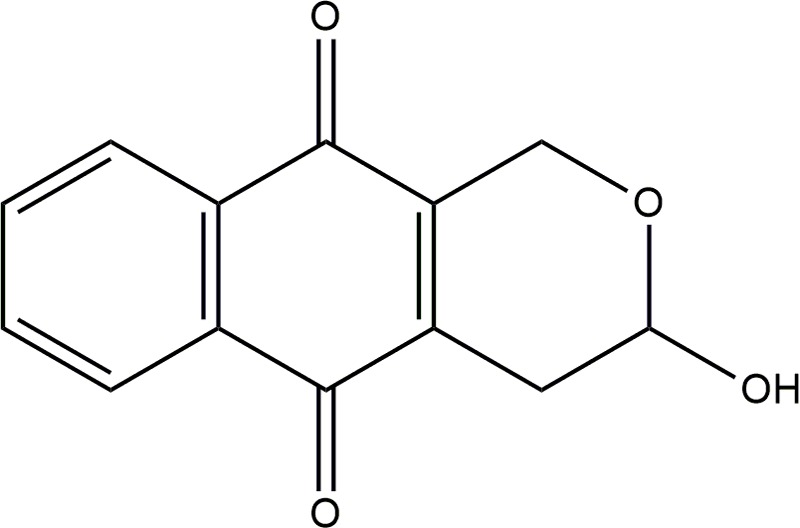
Chemical structure of psychorubrin.

Psychorubrin (**Figure 1**) has been associated with diverse biological activities, such as antitumoral (Hayashi et al., 1987; Fabri et al., 2012), cytotoxic (Hayashi et al., 1987), leishmanicidal (Fabri et al., 2012), and antiplasmodial (Endale et al., 2012) actions. In a previous work, we isolated psychorubrin from *Mitracarpus frigidus* (Fabri et al., 2009), a native species of Brazil (Pereira et al., 2006) and identified a promising potential antimicrobial property for this compound (Fabri et al., 2012). Here, the planktonic antibacterial activities of psychorubrin were investigated in detail as well as the potential bacterial biofilm control by this agent. By using different approaches, we found a consistent antagonistic activity for psychorubrin, which affects both bacterial forms and biofilms of Gram-positive bacteria, especially the multidrug-resistant *S. aureus* 33591 and 33592 in addition to *S. pyogenes* 10096. We also identified that the antimicrobial action of psychorubrin is linked with disruption of the bacterial cell membrane structure.

## Materials and Methods

### Plant Material

The aerial parts of *Mitracarpus frigidus* (Willd. Ex Roem. & Schult.) K. Shum (Rubiaceae) were collected in Juiz de Fora, Minas Gerais State, Brazil, in May 2011. The plant was identified by Dr. Tatiana Konno from the Ecological and Socio environmental Core of Macaé/UFRJ. A voucher specimen (CESJ 46076) was deposited at the Leopoldo Krieger Herbarium at the Federal University of Juiz de Fora (Brazil).

### Extraction and Reisolation

A crude dichloromethane (CH_2_Cl_2_) extract was prepared from *Mitracarpus frigidus* as before (Fabri et al., 2009). The isolation and structural elucidation of psychorubrin were performed as previously described in Fabri et al. (2012).

### Microbial Strains

The psychorubrin extract was screened by serial dilution assay against the following Gram-positive microorganisms: *Staphylococcus aureus* ATCC 25923, *Staphylococcus aureus* ATCC 33591 (Gentamicin and methicillin-resistant *Staphylococcus aureus* – MRSA), *Staphylococcus aureus* ATCC 33592 (Methicillin-resistant *Staphylococcus aureus* – MRSA) and *Streptococcus pyogenes* ATCC 10096 as well as the Gram-negative microorganisms *Escherichia coli* ATCC 10536, *Klebsiella pneumoniae* ATCC 4532 and *Pseudomonas aeruginosa* ATCC 9027. For subsequent tests, only *S. aureus* ATCC 33591, *S. aureus* ATCC 33592 and *S. pyogenes* ATCC 10096 were selected. The strains were cultured overnight at 37°C in Mueller Hinton agar (MHA) before each experiment.

### Serial Dilution Assay for Determination of the Minimal Inhibitory Concentration (MIC)

The minimal inhibitory concentration (MIC) of psychorubrin was determined by microdilution techniques in Mueller Hinton broth (MHB) as described (CLSI, 2014). Bacteria were cultured at 37°C for 24 h in MHA. The psychorubrin stock solution was twofold diluted ranging from 320 to 2.5 μg mL^-1^ (final volume = 80 μL) with final dimethyl sulfoxide (DMSO) concentration ≤ 1%. Then, 100 μL of MHB and 20 μL of 10^8^ CFU mL^-1^ (according to McFarland turbidity standards) bacterial suspensions were inoculated onto microplates, and the test was performed in a 200 μL final volume. Plates were incubated at 37°C for 24 h. Experiments were performed simultaneously for bacterial growth control (MHB + bacteria + psychorubrin vehicle) and sterility control (MHB + psychorubrin vehicle) as well as for the positive controls with chloramphenicol (100–0.2 μg mL^-1^) and ciprofloxacin (500–0.24 μg mL^-1^). MIC values were calculated from the highest dilution showing a complete inhibition of the tested strain. Analyses were done in triplicate. Compounds were considered significantly active when the MIC values were ≤ 10 μg mL^-1^(Kuete, 2017).

### Studies of Planktonic Bacteria

#### Bacterial Growth Curve

Psychorubrin was tested to determine the time-kill kinetics of strains. Saline suspensions of freshly grown bacterial strains (10^8^ CFU mL^-1^) were inoculated with different concentrations of psychorubrin (MIC, 0.5MIC, and 0.25MIC values) supplemented with MHB. Optical density (OD) at 600 nm was recorded in a spectrophotometer (Multiskan Go, Thermo Scientific, Waltham, MA, United States) at 2, 4, 6, 8, and 24 h of bacterial growth at 37°C as before (Pinto et al., 2017a,b). Graphs of turbidity versus incubation time were plotted. As a positive control, chloramphenicol was added. For bacterial growth control, groups consisting of MHB medium containing psychorubrin vehicle plus strains were used. Experiments were performed in triplicate (Babii et al., 2016).

#### Bacterial Abundance

Slides prepared in a cytocentrifuge were used to quantitate bacteria as before (Silva et al., 2014). Bacterial strains maintained in saline were inoculated into MHB containing psychorubrin (MIC value) and incubated at 37°C during 24 h. As a positive and negative control for this assay, chloramphenicol and psychorubrin vehicle at MIC values were added, respectively, to the groups containing bacterial strains. After diluting 10 times (1 mL) in saline, samples were fixed with sterile free-particle 37% formaldehyde to a final concentration of 2%, stained with 4′,6-Diamidino-2-Phenylindole (DAPI) (Vector Laboratories, Burlingame, CA, United States) for DNA recognition (final concentration of 0.01 μg mL^-1^) and placed in mega funnels (Shandon Mega Funnel, Thermo, United Kingdom) for immediate centrifugation in a cytocentrifuge (Shandon Cytospin 4, Thermo, United Kingdom), at 452 *g* at high acceleration during 10 min. Acceleration and speed were determined as the Cytospin manufacturer manual. Cells were analyzed under a fluorescence microscope (BX-60, Olympus, Melville, NY, United States) with a U-MWU2 filter (330–385 nm excitation wavelengths). For bacteria quantification, 20 random fields were counted for each sample at 1,000x magnification using an ocular grid. By applying a dilution factor (10x), the total bacterial count was obtained.

#### Bacteria Viability

Cell membrane integrity was investigated by using a fluorescent probe as an indicator for cell viability (Boulos et al., 1999). Bacterial samples (10 fold-diluted) were stained with the BacLight viability kit (Molecular Probes, Inc, Thermo Fisher Scientific, Eugene, OR, United States) and the proportion of live/viable and dead/non-viable cells was determined. This kit contains a mixture of fluorescent dyes, SYTO^®^ 9 and propidium iodide, which differ both in their spectral features and their ability to penetrate healthy bacterial cell membranes. Cells with intact membranes (live cells) stain green and those with damaged membranes (dead cells) stain red (Joux and Lebaron, 2000; Silva et al., 2014). Saline bacterial strains were inoculated into MHB containing psychorubrin at MIC value and incubated at 37°C during 24 h. Bacterial strains inoculated into MHB with psychorubrin vehicle or incubated with chloramphenicol (MIC values) served as negative and positive controls, respectively. For bacterial staining, samples were mixed with Baclight (1 mL of each sample to 3 μL of BacLight), placed in megafunnels (Shandon Mega funnel, Thermo, United Kingdom), cytocentrifuged as above and evaluated under a fluorescence microscope (BX-60, Olympus, Melville, NY, United States) at 450–480 nm excitation wavelengths (U-MWB filter) for simultaneous imaging of live and dead cells. For each sample, bacteria were directly counted in 10 random fields using an ocular grid at 1,000x magnification, and the average percentage of live/dead bacteria was obtained for each slide sampled.

#### Nucleotide Leakage

Nucleotide release was evaluated as before (Tang et al., 2008) with some modifications. Overnight bacterial cultures at 37°C were washed and resuspended in 10 mM PBS (pH 7.4), reaching a final density of nearly 10^8^ cells mL^-1^. Strains were then incubated with psychorubrin at MIC value during different times (2, 4, 6, and 8 h) while strains incubated with 10 mM PBS (pH 7.4) served as control groups. After incubation, cell suspensions were centrifuged at 10,000 *g* for 10 min; the supernatants were diluted appropriately, and the OD at 260 nm was recorded in a spectrophotometer (Multiskan Go, Thermo Scientific, Waltham, MA, United States) at room temperature (25°C). Experiments were performed in triplicate.

#### Checkerboard Test

The potential synergistic activity of psychorubrin and chloramphenicol was investigated by the checkerboard test (Pillai et al., 2005), which enables calculation of the fractional inhibitory concentration (FIC) index (FICI), that is the sum of the FICs (*Σ*FIC) of both agents. FICI values were interpreted as follows: FICI ≤ 0.5 synergy; 0.5 < FICI ≤ 1 additivity; 1 < FICI ≤ 2 indifference or no effect; and FICI ≥ 2 antagonism (Božić et al., 2014). The MIC of the two assayed agents was used for the FICI tested dilutions.

Assays were done using 96-well polystyrene microtiter plates with MHB, psychorubrin and chloramphenicol in twofold serial concentrations. Bacterial suspensions were prepared at a final concentration of 10^8^ CFU mL^-1^, incubated overnight at 37°C and read in a spectrophotometer (Multiskan Go, Thermo Scientific, Waltham, MA, United States). Tests were done in triplicate.

### Biofilm Studies

#### Effect on Established Biofilms

The effect of psychorubrin on established biofilms was tested as before (Nostro et al., 2007) with some alterations. Briefly, biofilms of *S. aureus* 33591, *S. aureus* 33592 and *S. pyogenes* 10096 strains were generated with each of these organisms using 96-well polystyrene microtiter plates filled with MHB, 1% glucose and cells (conc 10 cells^7^/mL) during 24 h at 37°C. Then, the planktonic cells were gently removed and, after washing with saline (three times), the wells were filled with 200 μL of psychorubrin (twofold dilutions), with MIC in the range of four-fold dilution. Incubation was performed at 37°C during 24 h. OD was evaluated at 492 nm at time 0 and at 24 h after incubation. As a positive control for this assay, chloramphenicol was added. For biofilm growth control, groups consisting of MHB medium plus strains were used. All experiments were performed in triplicate. To calculate the percentage of biofilm inhibition, the OD values of the growth control group for each strain were compared with that of the treated group using the following equation:

$$\begin{array}{c} {}[(\mathrm{OD}(\mathrm{control})–\mathrm{OD}(\mathrm{treatment})/OD(\mathrm{control})]\;\times \;100 \end{array}$$

#### Effect on Adherence of Biofilms

Inhibition of biofilm formation was performed by spectrophotometric assay according to (Plyuta et al., 2013). Cell suspensions (100 μL) of *S. aureus* 33591, *S. aureus* 33592 and *S. pyogenes* 10096 (10^8^ CFU/mL) and different concentrations of psychorubrin and chloramphenicol (MIC, 0.5 MIC, and 0.25 MIC) were incubated at 37°C during 24 h. The suspensions were then removed, and the wells were washed with 200 μL of PBS to remove free-floating bacteria. Biofilms formed by adherent cells in the plate were stained with 200 μL of 0.1% crystal violet and incubated at room temperature for 30 min. Excess stain was rinsed off by thorough washing with PBS, plates were fixed with 200 μl of 96% ethanol and incubated for 15 min. The resulting reaction was read spectrophotometrically at 590 nm. All experiments were performed in triplicate. The percentage of biofilm inhibition was calculated using the following equation:

$$\begin{array}{c} {}[(\mathrm{OD}(\mathrm{control})–\mathrm{OD}(\mathrm{treatment})/OD(\mathrm{control})]\;\times \;100 \end{array}$$

#### Effect on Biofilm Matrix Composition

The biochemical composition of the biofilm matrix from *S. aureus* 33591, *S. aureus* 33592, and *S. pyogenes* 10096 was studied by using the bicinchoninic acid method for proteins (Smith et al., 1985) and the phenol-sulphuric acid method for total carbohydrates (Dubois et al., 1956). Briefly, adherent biofilms were transferred to screw cap bottles containing 2 mL of distilled water. The bottles were sonicated for 5 min in an ultrasonic water bath and vortexed vigorously for 1 min to disrupt the biofilms. Cell suspensions were then pooled, centrifuged and the supernatants collected for subsequent evaluation of the total content of proteins and carbohydrates.

#### Scanning Electron Microscopy (SEM)

*Staphylococcus aureus* 33591, *S. aureus* 33592, and *S. pyogenes* 10096 strains were seeded in MH agar, incubated for 24 h at 37°C, and inoculated into a tube containing 5 mL of BHI broth with 1% glucose. Then, 500 μL of the inoculated broth (10^8^ UFC mL^-1^) was added in plated of 24 wells containing round glass coverslips (13 mm, Glasscyto^®^). The treatment (*n* = 3 wells) was done by adding 500 μL of the psychorubrin (final concentration per well = MIC). For negative (*n* = 3) and positive (*n* = 3) controls 500 μL of sterile water or chloramphenicol (MIC) were added, respectively. Biofilms on glass coverslips (13 mm, Glasscyto^®^) were cultured for 24 h at 37°C and fixed in 2.5% glutaraldehyde for 30 min at room temperature (Riedel-de-Haen, Germany) in 0.1 M cacodylate buffer (pH 7.2). Coverslip-adherent cells were post-fixed with osmium tetroxide, and dehydrated through a graded series of ethanol solutions (30, 50, 70, 90% and twice in 100%) for 15 min at each concentration. Cells were then critical point dried in carbon dioxide. Coverslips were mounted on aluminum holders, sputtered with 5 nm gold, and analyzed in a scanning electron microscope (JEOL JSM-6390LV, Tokyo, Japan) for observation of the biofilms and bacterial morphology.

### Statistical Analysis

Results are expressed as mean values with the standard error. The statistical analyses were performed using ANOVA test followed by Bonferroni to compare the controls and treated groups at a significance level of 5%

## Results

### Minimal Inhibitory Concentration (MIC)

Psychorubrin showed a broad spectrum of antibacterial activity, with clear effectiveness against both Gram-negative and Gram-positive bacteria. However, the three bacteria species most susceptible to psychorubrin according to MIC values were *S. aureus* 33591, *S. aureus* 33592, and *S. pyogenes* 10096, in this order (**Table 1**). Psychorubrin was more effective than chloramphenicol and less effective than ciprofloxacin for *S. aureus* 33591, *S. aureus* 33592, and *S. pyogenes* 10096. Based on these observations, these three bacteria strains were selected for further investigation of the psychorubrin activity.

**Table 1:** Antibacterial activity of the psychorubrin.

	PSY	CHL	CIP
	
	MIC^∗^	MIC^∗^	MIC^∗^
*Staphylococcus aureus* 25923	80	12.5	2.5
*Staphylococcus aureus* 33591	5	25	0.16
*Staphylococcus aureus* 33592	5	25	0.08
*Streptococcus pyogenes* 10096	10	12.5	0.63
*Klebsiella pneumoniae* 4352	20	0.78	0.31
*Escherichia coli* 10536	20	3.13	0.08
*Pseudomonas aeruginosa* 9027	160	25.0	0.63

*PSY, psychorubrin; CHL, chloramphenicol; CIP, ciprofloxacin; ^∗^Values in μg mL^-*1*^.*

### Bacterial Killing Assay

**FIGURE 2:**
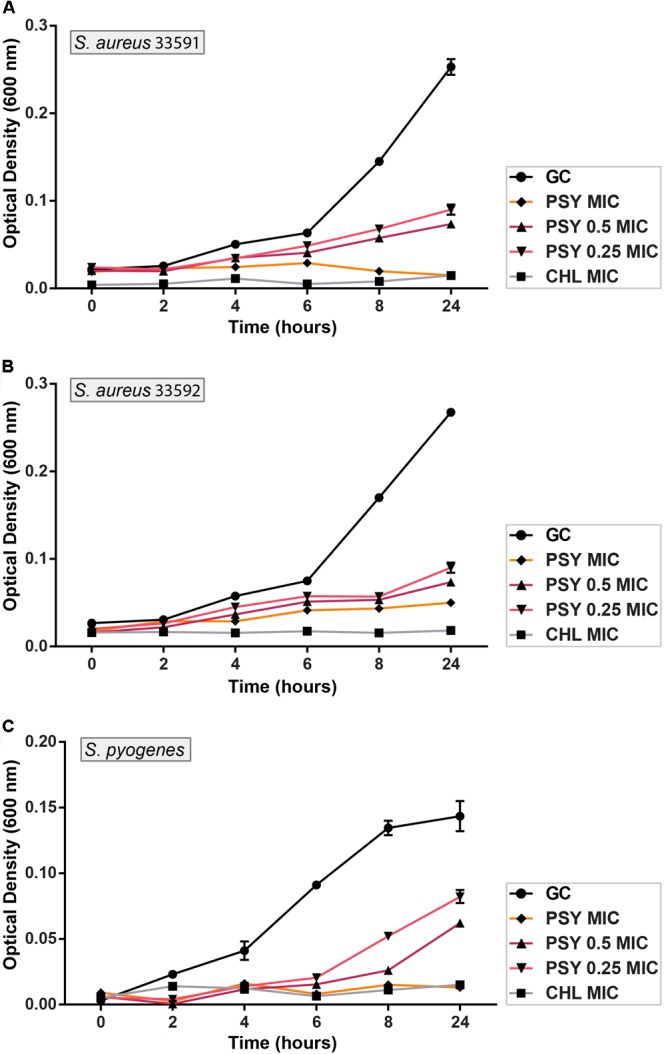
Activity kinetics of psychorubrin (PSY) against *S. aureus* 33591 **(A)**; *S. aureus* 33592 **(B);** and *S. pyogenes* 10096 **(C)**. Chloramphenicol (CHL) was used as positive control, and bacteria inoculated in MHB with psychorubrin vehicle was used as bacteria growth control (GC). The experiments were carried out in triplicate, and data represent the mean ± SD.

The psychorubrin vehicle did not affect the bacterial growth curve for all species. ON the other hand, all tested bacteria showed a dose-dependent growth decrease when exposed to psychorubrin (**Figure 2**). All the concentrations evaluated (MIC, 0.5 MIC, and 0.25 MIC) inhibited the growth cycle curve of the three tested bacteria in comparison to the vehicle-treated control. For *S. aureus* 33591 and *S. pyogenes* 10096 (**Figures 2A,C**) psychorubrin extended the lag phase by 4 h (0.5 MIC and 0.25 MIC), and inhibited fully bacterial growth at MIC value similar to chloramphenicol. For *S. aureus* 33592, all concentration for psychorubrin extended the phase lag by 2 h (**Figure 2B**).

### Bacterial Abundance and Viability

**FIGURE 3:**
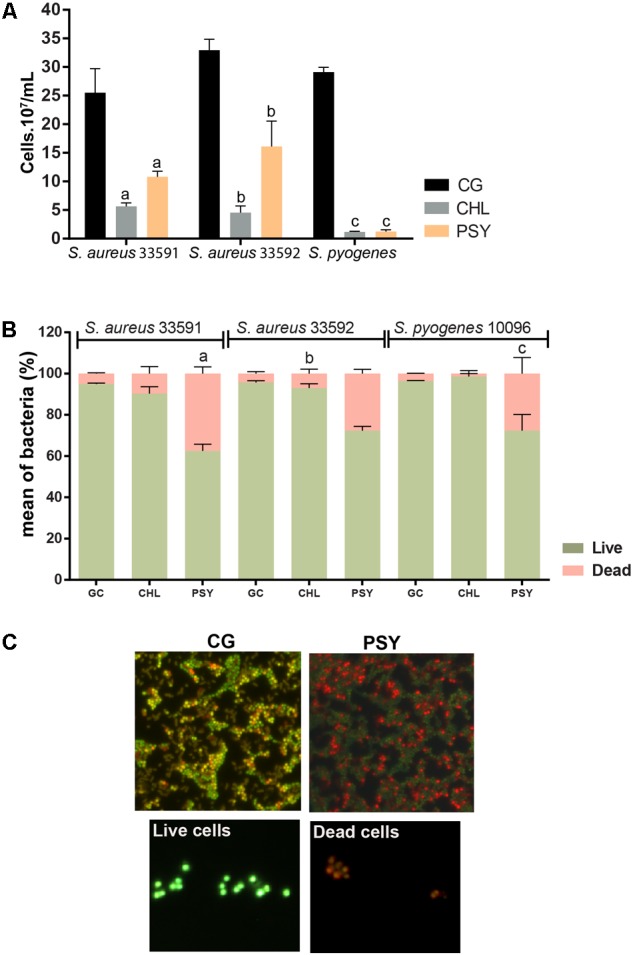
Effect of psychorurin (PSY) on the growth of *S. aureus* 33591, *S. aureus* 33592 and *S. pyogenes* 10096. Cultured bacteria were stained with DAPI or LIVE/ DEAD^®^ BacLight^TM^ and counted under fluorescence microscopy for evaluation of cell density **(A)** and viability **(B,C)**, respectively. Psychorubrin treatment induced both decrease of cell density **(A)** and increase of cell death **(B)** compared to control groups treated with psychorubrin vehicle. **(C)** Representative images from *S. aureus* 33501 show dead cells in red and live cells in green after baclight staining. Letters indicate statistical difference from controls treated with psychorubrin vehicle for *S. aureus* 33591 (^a^), *S. aureus* 33592 (^b^), *S. pyogenes* 10096 (^c^) (ANOVA followed by Bonferroni, *P* < 0.05). Chloramphenicol (CHL) was used as a positive control. All experiments were carried out in triplicate, and data represent the mean ± SD of bacteria counted.

Fluorescence microscopy after DAPI staining showed that psycorubrin inhibited the bacterial abundance compared to the control group, reducing bacterial growth by 52, 50, and 96% for *S. aureus* 33591, *S. aureus* 33592 and *S. pyogenes* 10096, respectively (**Figure 3A**). Psychorubrin treatment produced decrease of cell density and increase of cell death.

The cytocentrifuge preparations of the cell viability probe test (Live/Dead^®^ BacLight) revealed the presence of live (green) and dead (red) bacteria in all psychorubrin-treated and control groups (**Figures 3B,C**). Psychorubrin treatment (MIC value for 24 h) led to an increase of the dead cell numbers for *S. aureus* 33591, *S. aureus* 33592, and *S. pyogenes* 10096, corresponding to 37, 28, and 28% of dead cells, respectively, in comparison to the vehicle-treated group (*S. aureus* 33591 = 94.91%, *S. aureus* 33592 = 95.68%, *S. pyogenes* 10096 = 96.40%). Chloramphenicol showed 9, 7, and 1.3% of dead cells, respectively.

### Nucleotide Leakage

**FIGURE 4:**
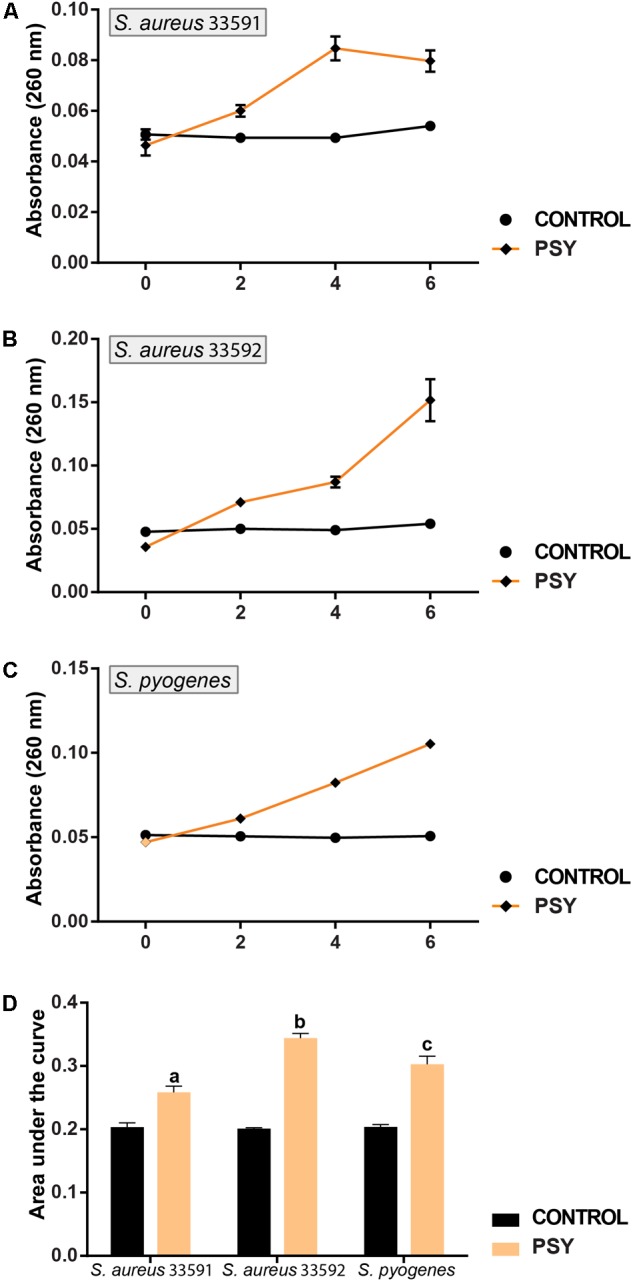
Mode of action of psychorubrin (PSY). Effect of psychorubrin on the amount of the total nucleotide released from *S. aureus* 33591 **(A)**, *S. aureus* 33592 **(B)**, and *S. pyogenes* 10096 **(C)**. Area under the curve in PSY-treated and controls are showed in **(D)**. Letters indicate statistical difference from controls treated with psychorubrin vehicle for S. aureus 33591 (^a^), *S. aureus* 33592 (^b^), *S. pyogenes* 10096 (^c^) (ANOVA followed by Bonferroni, *P* < 0.05). All experiments were carried out in triplicate, and data represent the mean ± SD.

The efflux of nucleotides from the intracellular compartment was significant for the three Gram-positive bacteria. As shown in (**Figures 4A–D**) psychorubrin increased at 19, 41, and 30% the nucleotide release in *S. aureus* 33591, *S. aureus* 33592, and *S. pyogenes* 10096 respectively, compared to the controls.

### Checkerboard

The checkerboard assay showed a decrease in the MIC values for both psychorubrin and chloramphenicol, suggesting possible interactions between these substances. The results demonstrated a synergistic action between psychorubrin and chloramphenicol for *S. aureus* 33592 and *S. pyogenes* 10096, and an additive effect between psychorubrin and chloramphenicol for *S. aureus* 33591 (**Table 2**).

**Table 2:** Fractional inhibitory concentration (FIC) indices.

Microorganisms	PSY	CHL	ΣFIC	Effect
			
	MIC^∗^	MIC^∗^		
*Staphylococcus aureus* 33591	0.078	12.5	0.52	Additive
*Staphyloccocus aureus* 33592	0.078	3.13	0.14	Synergy
*Streptococcus pyogenes* 10096	0.312	1.95	0.14	Synergy

*PSY, psychorubrin; CHL, chloramphenicol; ^∗^MIC in a combination of psychorubrin and chloramphenicol (μg/mL). *Σ*FIC: sum of fractional inhibitory concentrations – the combinatory effect is evaluated as follows: synergy *Σ*FIC ≤ 0.5; additive *Σ*FIC > 0.5 and ≤ 1; indifferent *Σ*FIC > 1 and ≤ 2.*

### Effect on Established Biofilms and on Their Adhesion

**FIGURE 5:**
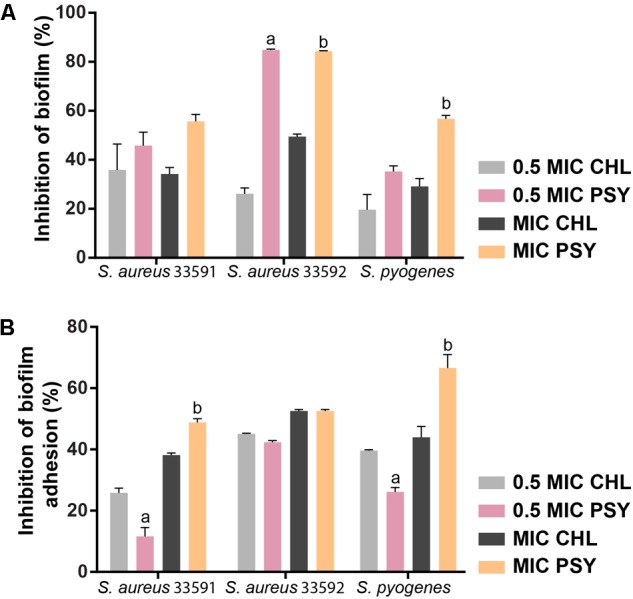
The effect of psychorubrin (PSY) on established biofilms **(A)** of *S. aureus* 33591, *S. aureus* 33592, and *S. pyogenes* 10096 and on their adherence ability **(B)**. Choramphenicol (CHL) was used as positive control. Each data point represents mean ± SD from at least two independent experiments performed in triplicate. ^a^Statistically different from chloramphenicol (MIC value). ^b^Statistically different from chloramphenicol (0.5 MIC value) (ANOVA followed by Bonferroni, *P* < 0.05).

To investigate if psychorubrin was able to disrupt pre-formed biofilms and to affect their adhesion capability, samples were evaluated at the MIC and 0.5MIC (**Figure 5**). The disruption and adhesion of biofilm at MIC and 0.5MIC were significantly effective when compared to positive controls. In these concentrations, the inhibition of pre-formed biofilms of psychorubrin was about 56 and 46% for *S. aureus* 33591, 84 and 85% for *S. aureus* 33592, 57 and 35% for *S. pyogenes* 10096, respectively. Psychorubrin also decreased the adhesion of biofilms with percentages of inhibition of 49 and 12% for *S. aureus* 33591, 52 and 42% for *S. aureus* 33592, 67 and 26% *S. pyogenes* 10096, respectively with MIC and 0.5MIC values.

### Effect on Biofilm Matrix Composition

**Table 3** shows the total content of carbohydrates and proteins of formed biofilms by *S. aureus* 33591, *S. aureus* 33592 and *S. pyogenes* 10096. Psychorubrin and chloramphenicol (MIC values) significantly reduced carbohydrate and protein contents, being the maximum reduction observed for total content of proteins.

**Table 3:** Biochemical composition of biofilms treated with psychorubrin and chloramphenicol.

Microorganims	GBC		PSY		CHL	
	TP	TC	TP	TC	TP	TC
*Staphylococcus aureus* 33591	145.1 ± 0.2	0.628 ± 0.02	1.2 ± 0.01	0.148 ± 0.01	5.3 ± 0.07	0.077 ±0.001
*Staphyloccocus aureus* 33592	153.7 ± 0.7	0.654 ± 0.01	6.7 ± 0.02	0.148 ± 0.03	7.0 ± 0.1	0.106 ± 0.005
*Streptococcus pyogenes* 10096	67.2 ± 0.5	0.624 ± 0.03	0.2 ± 0.01	0.135 ± 0.04	1.0 ± 0.001	0.104 ± 0.06

*GBC, growth biofilm control; PSY, psychorubrin; CHL, chloramphenicol; TP, total protein in μg mL^-*1*^; TC, total carbohydrate in *μ*g mL^-*1*^.*

### SEM Observations

**FIGURE 6:**
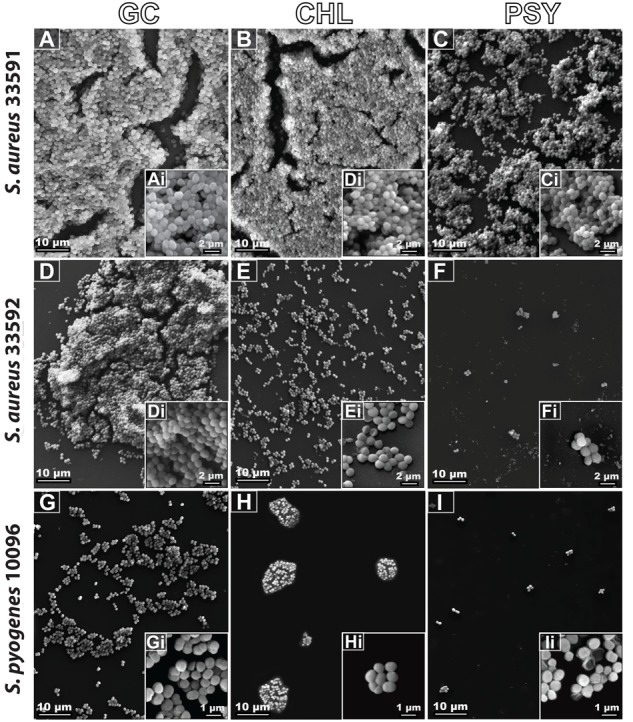
Biofilm scanning electron micrographs of the different bacterial strains with or without antimicrobial treatment. GC, growth control **(A,D,G)**; CHL, chloramphenicol **(B,E,H)**; PSY, psychorubrin **(C,F,I)**. Note in high magnification (inset), the morphological external structure of the different bacterial strains.

Ultrastructural analyses by SEM revealed the surface structure and morphology of biofilms formed by different bacterial strains with or without antimicrobial treatment (**Figure 6**). The control group showed the typical multilayer growth of bacterial biofilms (**Figures 6A,D,G**) while the group treated with chloramphenicol exhibited a significant reduction of biofilms only for the *S. aureus* 33592 and *S. pyogenes* 10096 strains (**Figures 6B,E,H**). Remarkably, psychorubrin led to a significant reduction of biofilm formation in all strains (**Figures 6C,F,I**), with loss of the bacterial original shape as evidenced by a distorted and irregular bacterial cell wall (**Figure 6I**). Altogether, our findings provide evidence that psychorubrin has a potent antimicrobial action against the different strains analyzed.

## Discussion

New antimicrobial compounds are greatly needed to treat infections caused by bacteria resistant to currently available agents. Here, we provided direct evidence that the bioactive compound psychorubrin has a broad spectrum of antibacterial activity being effective against both bacterial forms and biofilms of Gram-positive bacteria. We demonstrated a strong inhibitory activity of this agent for *S. pyogenes* and *S. aureus* including multiresistant strains. Therefore, the present study expands previous findings (Fabri et al., 2012) highlighting the use of psychorubrin as a possible strategy to treat the infections caused by these microorganisms.

The time-kill curves can monitor bacterial growth and death over a wide range of antimicrobial concentrations and have been frequently used to evaluate the effect of antimicrobials over time (Foerster et al., 2016). A time-dependent bactericidal effect happens when the concentration of the antibacterial drug surpasses the MIC for the microorganism, while the concentration-dependent bactericidal effect takes place when an antibiotic has a high concentration at the binding site to eradicate the microorganism (Anantharaman et al., 2010). Our time-kill kinetics analyses showed that psychorubrin presented a rapid time-dependent kinetics of bacterial killing for all tested bacteria, mainly at MIC value. Comparing the activity of the psychorubrin with chloramphenicol at MIC concentration, we observed a similar action starting at initial hours (**Figure 2**).

Psychorubrin prolonged the lag phase for the three tested bacteria (*S. aureus* 33591, *S. aureus* 33592, and *S. pyogenes* 10096) with 0.5 MIC and 0.25 MIC values. Since the lag phase is a response during the adaptation period by first division of the bacterial cell, this process could involve the repair of macromolecular damage that occurred in the ambient that the cell came from and the synthesis of cellular components necessary for growth (Baty and Delignette-Muller, 2004; Rolfe et al., 2012). Therefore, one of the possible psychorubrin targets might be the synthesis of macromolecules, since pyranonaphthoquinones and naphthoquinones can act in the redox cycle generating a pro-oxidant state that favors the occurrence of oxidative lesions in macromolecules and cellular structures thus resulting in cell death (Silva et al., 2003). In fact, we found a efficient inhibition of bacterial growth by psycorubrin during 24 h indicating a bactericidal effect of this compound, especially for *S. pyogenes* 10096. Moreover, the efficiency of psychorubrin was a little better than chloramphenicol.

To investigate the effect of psychorubrin on bacterial density and viability, we performed two additional approaches. By using fluorescent probes for DNA (DAPI staining) and membrane integrity (*Backlight*) (Gamalier et al., 2017), we showed that the psychorubrin treatment was able to reduce cell density and increase cell death, thus corroborating our bacterial killing assay results. The psychorubrin-elicited bacterial death may be related with a potential inhibitory effect of this compound on bacterial growth and/or a direct effect on bacterial cells, leading to cell death. Because the marker for cell viability enables the detection of compromised plasma membranes, dead cells can be directly identified before complete cell lysis (Gamalier et al., 2017). Indeed, when cells exhibiting damaged membranes are not able to keep an electrochemical potential, they are considered dead (Joux and Lebaron, 2000). Thus, our approach was helpful to recognize bacterial viability at a single-cell level (Joux and Lebaron, 2000; Silva et al., 2014; Gamalier et al., 2017).

The significant increase of dead cells induced by psychorubrin is in accordance with previous studies, which demonstrated bactericidal activity after treatment with compounds through viability analyses using *Baclight* (You et al., 2013; Yuan and Yuk, 2018). Additionally, the bacteriostatic effect from chloramphenicol (Bernatová et al., 2013), showed a very low proportion of dead cells, statistically different from psychorubrin. Overall, our viability results support the potential bactericidal effect of psychorubrin for the three bacteria tested, indicating the occurrence of membrane damage. Therefore, the microscopic approaches used in the present study were especially revealing in demonstrating the mode of action of psychorubrin.

Our results showed that psychorubrin is effective against Gram-positive bacteria, but the antimicrobial activity is organism-dependent. In this context, nucleotide leakage assay was used to clarify how psychorubrin is able to inhibit proliferation of these bacteria (Hao et al., 2009). As DNA and RNA are released after membrane disruption, these nucleotides were quantified by monitoring the absorbance at 260 nm. The results of nucleotide leakage assay confirm the occurrence of membrane destabilization triggered by psychorubrin (**Figure 4**).

Considering antimicrobial therapy, drug combination has many advantages compared with the use of individual agents. It can be used to prevent the emergence of resistant organisms, to minimize toxicity due to the need of lower drug concentrations, and to obtain synergistic antimicrobial activity (Ocampo et al., 2014). The association of psychorubrin and chloramphenicol was important to optimize the antimicrobial effect of both. However, future studies are needed to test antimicrobial resistance to other clinical choice drugs.

One interesting observation from the present study was the efficiency of psychorubrin in disrupting pre-formed biofilms and decreasing biofilm adhesion for different bacterial species (**Figure 5**). These results combined with our findings showing the psychorubrin effect on biofilm matrix composition, indicate that this compound leads to the inhibition of exopolysaccharide synthesis and especially to the interference of protein formation. Altogether, our data demonstrated that psychorubrin limited the formation of biofilm. Considering that the matrix exopolysaccharide is one of the most distinctive features of a microbial biofilm, forming a three dimensional, gel-like, highly hydrated and locally charged environment in which the microorganisms are largely immobilized (Namasivayam and Roy, 2013), biofilm reduction must be considered the first step for its control by an antimicrobial agent. Furthermore, the restriction of the biochemical composition of the biofilm matrix leads to weakening of the biofilm thus facilitating the entry of the drugs (Joseph, 2003). Our SEM analyses reinforced the action of psychorubrin over the strains tested, both in biofilm form reducing the multilayer growth, and free living cells by affecting the integrity of cell wall.

## Conclusion

Taken together, our findings identify, for the first time that psychorubrin is active against all tested Gram-positive and Gram-negative bacteria, especially *S. pyogenes* and *S. aureus*, including multi-resistant strains. Considering these two species, this compound has a bactericidal effect, targeting the bacterial synthesis of macromolecules and rapidly (2 h) destabilizing their membranes. The efficacy of psychorubrin showed to be better than chloramphenicol for *S. pyogenes* 10096 and the association of these agents showed at least an additive effect. The bioactivities of psychorubrin identified in this study include both planktonic and biofilm growing Gram-positive bacteria. Psychorubrin was able to reduce bacteria biofilms by interfering with protein formation and inhibiting exopolysaccharide synthesis. These results are promising. Given the potential of psychorubrin as an antibiofilm drug against *S. aureus* and *S. pyogenes*, and the possible combination with chloramphenicol, this compound could be used to reduce the development of resistance to these microorganisms and improve the outcome of the therapies for infections caused by these pathogens. This is of great relevance in hospital services, especially considering the multi-resistance of MRSA.

## Author Contributions

AL, LC, AA, and RF designed the study. AL, LC, TS, LO, MG, and VR performed the field work and laboratory work. RM, JA, AA, and RF analyzed the data. RM, VR, ES, and RF wrote the manuscript. All authors reviewed the manuscript.

## Conflict of Interest Statement

The authors declare that the research was conducted in the absence of any commercial or financial relationships that could be construed as a potential conflict of interest.
